# Mitophagy Modulation, a New Player in the Race against ALS

**DOI:** 10.3390/ijms22020740

**Published:** 2021-01-13

**Authors:** Enrique Madruga, Inés Maestro, Ana Martínez

**Affiliations:** 1 Centro de Investigaciones Biológicas-CSIC, Ramiro de Maeztu 9, 28040 Madrid, Spain; enrique.madruga@cib.csic.es (E.M.); ines.maestro@cib.csic.es (I.M.); 2 Centro de Investigación Biomédica en Red de Enfermedades Neurodegenerativas (CIBERNED), Instituto de Salud Carlos III, 28031 Madrid, Spain

**Keywords:** amyotrophic lateral sclerosis, mitophagy, mitochondria, drug target

## Abstract

Amyotrophic lateral sclerosis (ALS) is a lethal neurodegenerative disease that usually results in respiratory paralysis in an interval of 2 to 4 years. ALS shows a multifactorial pathogenesis with an unknown etiology, and currently lacks an effective treatment. The vast majority of patients exhibit protein aggregation and a dysfunctional mitochondrial accumulation in their motoneurons. As a result, autophagy and mitophagy modulators may be interesting drug candidates that mitigate key pathological hallmarks of the disease. This work reviews the most relevant evidence that correlate mitophagy defects and ALS, and discusses the possibility of considering mitophagy as an interesting target in the search for an effective treatment for ALS.

## 1. Introduction

Amyotrophic lateral sclerosis (ALS) is a lethal neurodegenerative disease that affects motoneurons, both superior (those that arise from the cortex and innervate the spinal cord and the brain stem) and lower (those that arise from the spinal cord, brain stem, and innervate muscular tissues) [1]. Similar to other neurodegenerative diseases, ALS is considered a multifactorial proteinopathy that currently has no effective treatment available. According to the ALS Therapy Development Institute, around 450,000 people are living with ALS worldwide, a number that is expected to raise dramatically in the next years due to the increase of life expectancy, reaching a 69% growth in diagnoses globally by 2040 [2]. Nowadays, only riluzole and edavarone (US and Japan only) are approved for this devastating disease. Although these drugs show evidence of extended survival times in patients of a few months, this improvement is not enough to remedy the public health challenge. Therefore, discovering an effective treatment for ALS is highly needed.

ALS usually onsets in adult life, around 55–60 years [3]. Symptoms can vary depending on which motoneuron is affected, and patients usually suffer from weakness and muscular atrophy in limbs or dysphagia and dysarthria. Regardless of the onset of the pathology, there is usually a rapid progression of the disease that ends with the patient’s death in an interval of 2 to 4 years due to respiratory dysfunction [1]. Around 10% of ALS cases can relate to a genetic component of the disease, affecting individuals who have, at least, one family member that also manifests the pathology (familiar ALS, fALS). Almost all fALS cases are due to an autosomal dominant inheritance, however, only 50% of them can be associated with specific ALS-related genes, which mainly consist of *C9ORF72* (40%), *SOD1* (20%), *FUS* (1–5%), and *TARBDP* (1–5%, encoding for the canonical 43-kDa transactive response DNA binding protein, TDP-43) [4]. Furthermore, genotype does not necessarily predict the phenotype [5], and the same ALS-related mutation can lead to different clinical manifestations. The rest of the cases (90%) that cannot be related to family inheritance are considered sporadic (sporadic ALS, sALS).

**Figure 1:**
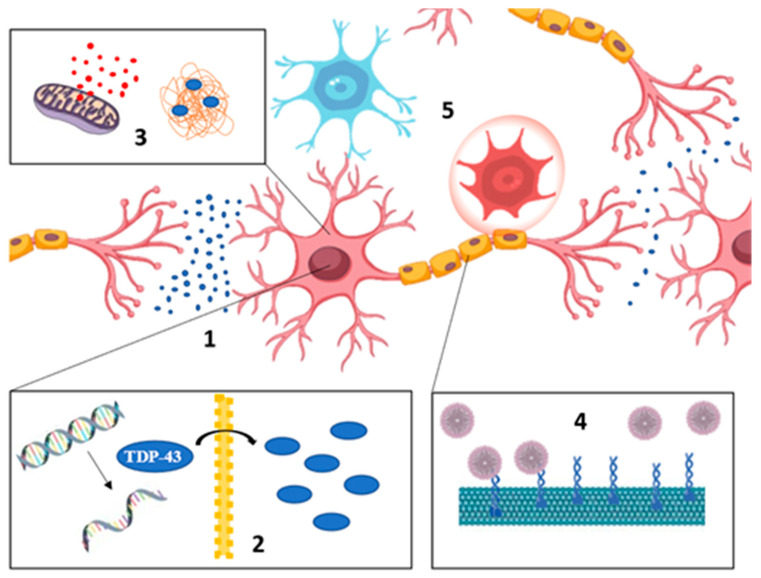
Main pathogenic pathways involved in amyotrophic lateral sclerosis (ALS). (**1**) Excitotoxicity due to an excessive glutamatergic activity, (**2**) impaired RNA metabolism, (**3**) accumulation of defective mitochondria and protein aggregates, (**4**) incorrect axonal transport, and (**5**) neuroinflammation.

Molecularly, most patients show motoneuron aggregates usually formed by misfolded, phosphorylated, ubiquitinated, or truncated proteins, among other modifications [3]. Cytoplasmatic TDP-43 aggregates can be found in 97% of ALS patients, while TDP-43 usually presents a nuclear location in physiological conditions [6]. Other patients show different kinds of protein aggregates such as superoxide dismutase 1 (*SOD1*) or fused in sarcoma protein (*FUS*). Neuroinflammation, excitotoxicity, axonal transport defects, and autophagy dysregulation are some of the manifestations that can also be observed during the disease onset (Figure 1). The extended length of the motoneuron axons and the need to continuously maintain homeostasis conditions may be the cause of the multitude fronts implicated in ALS [5]. Thus, elucidating the primary cause of ALS results is highly complex, as well as deciphering the molecular mechanisms that could be considered to find effective therapeutic targets.

Neurons display high energy and metabolic demand in comparison with other kinds of cells. They use 20% of the oxygen consumed by the human body, but account for only 2% of the weight of an average adult. This demand can fluctuate drastically, causing high levels of needed oxygen in short intervals of time that result in an increase in reactive oxygen species (ROS) levels [7]. This process leads to neuronal damage since neurons are dramatically sensitive to these reactive species, due to their low antioxidant capacity, high content of polyunsaturated fatty acids, and minimal regenerative capacity [8]. In addition, ROS can damage mitochondrial DNA, decreasing its ability to generate adenosine triphosphate (ATP) and cyclically increasing free radical levels. Moreover, neurons are highly polarized cells, where the potential of the different membranes plays a fundamental role in the proper functioning of the nervous system. Intra- and extracellular ion levels, such as Ca^2+^, K^+^, and Na^+^, must be tightly regulated. According to this, mitochondrion could be an interesting “master key” in ALS pathology due to their major role in ATP synthesis, ROS production, Ca^2+^ regulation, and apoptosis triggering [9,10,11]. Mitochondrial defects in ALS models have extensively been reported, from excessive ROS production that results in oxidative stress to low energy levels because of inadequate ATP synthesis, defective organelle morphologies, or an imbalance between mitochondrial biogenesis and degradation [12,13,14,15]. Indeed, mitochondrial accumulation has been found in the soma of motoneurons in the spinal cord of ALS patients [16]. Genetically, several ALS-related genes have been associated to autophagy or mitophagy defects in the last years. Although there are many questions that remain unsolved, there seems to be a relation between ALS and the accumulation of dysfunctional mitochondria. All the above highlight an exciting but hard-to-solve question: could mitophagy stimulation be the correct key to push in the search of an ALS treatment?

## 2. Mitochondrial Homeostasis in ALS

Every cell needs homeostasis conditions, a dynamic balance designed to maintain all its components, from ions to organelles, in proper conditions both in time and space. It includes a set of mechanisms in charge of degrading defective or overabundant elements. When this machinery fails to keep homeostasis, different kinds of aggregates may appear leading to disease in ALS cases. *SOD1* was the first protein observed to form insoluble aggregates in ALS pathology in 1993, and mainly found in fALS cases [17]. However, more importantly, around 97% of ALS patients show cytoplasmic TDP-43 aggregates, regardless of whether they have mutations in the gene that encodes it, *TARBDP,* which is present in only 5% of fALS cases. *FUS* aggregates are also found in the cytoplasm, but are not as widespread. Both TDP-43 and *FUS* proteins have predominantly nuclear location under physiological conditions but their pathological aggregates are found in the cytoplasm [18]. Curiously, *C9ORF72* mutations are found in 40% of fALS and these patients show not only TDP-43 aggregates, but also aggregates formed by dipeptide repeat proteins formed by repeat-associated, non-AUG-initiated translation [19]. The fact of whether toxicity is caused either by the aggregates themselves or by the high molecular weight complexes that precede them is still unclear. In any case, pathological accumulation of these proteins is a hallmark in ALS, therefore, their elimination is one of the most active strategies to find an effective ALS treatment.

On another note, ALS is also characterized by the accumulation of dysfunctional mitochondria. These organelles never act as discrete units, but form a highly dynamic network that undergoes multiple events. The organelle is sustained by means of fusion, fission, biogenesis, and degradation mechanisms according to cellular needs. Thus, maintaining a healthy pool of these organelles is important and drastic changes in mitochondrion morphology, mislocalization, or an inappropriate number of them are usually related to an inadequate mitochondrial homeostasis. Many pathological pathways involved in ALS can be related to mitochondrial defects one way or another: typical axonal transport defects in ALS impair mitochondria distribution [20]; altered Ca^2+^ homeostasis due to glutamate hyperexcitability involves mitochondria as the major Ca^2+^ storage site [21]; defective mitochondria can release proapoptotic factors that can cause neuronal death [22]; an increase in ROS levels due to mitochondrial metabolism leads to neuroinflammation in microglia [23]; among others. Thus, focusing on mitochondrion as a convergence point can clarify the complexity of ALS pathogenesis. Several mitochondrial defects in ALS have been published and many ALS-related genes are involved in mitochondrial dysfunction. Abnormal structures and metabolic functions in mitochondria have been found in the spinal cord of ALS patients [24]. *FUS*^R495X^ mutation shortens mitochondria and induces neurotoxicity [25]. Mutant *SOD1* binds mitochondrion and alters normal shape and distribution in mouse models [13] and impairs anterograde axonal transport of mitochondria through the reduction of mitochondrial Rho GTPase 1 (MIRO1) levels [26], a critical protein needed to transport mitochondria from the axon to the soma. TDP-43^A315T^ mice show elongated mitochondria in dendrites and structural defects in the inner membrane [27]. Peculiarly, there are many ALS-related genes whose mutations are implicated in an inefficient level of cell component degradation, including defective mitochondria. The relation between ALS genes and defective mitophagy is discussed below. Keeping in mind that mitochondrial defects appear at early stages of the disease, trying to ensure correct levels of healthy mitochondria in ALS is an interesting strategy to explore.

## 3. Autophagy and Mitophagy

Ubiquitin is a marker of components that must be degraded, and the accumulation of ubiquitin-positive aggregates may be a sign of a defective degradation mechanism. While there are several degradation pathways with different roles, all of them can be classified in (i) the ubiquitin-proteasome system (UPS), which is responsible for marking proteins by ubiquitination and subsequent degradation by the proteasome, and (ii) autophagy, whose role is most related to the degradation of proteins and organelles through lysosomes. Macroautophagy (henceforth referred to as autophagy) is one kind of autophagy responsible for the degradation of proteins and organelles, involving them in double-membrane vesicles termed autophagosomes. These vesicles end up merging with the lysosomes forming the autolysosomes [28,29]. Autophagy has usually been related to starving conditions, where a low level of nutrients would trigger this pathway to recycle the components the cell needs. Curiously, notable basal autophagy levels have been reported in neurons in the absence of starvation stimulus [30]. Neurons are prone to damage accumulation because of their postmitotic nature, so autophagy results are critical to maintain homeostasis. Mitochondria degradation through autophagy, usually termed as mitophagy, implies the total or partial degradation of the defective organelle with the consequent energy and time spent involved. Other systems like UPS or chaperones are the first line in mitochondrial quality control, and only when these previous systems cannot maintain the healthy pool of mitochondria is mitophagy triggered [10,31]. Misfolded proteins, membrane depolarization, a high level of ROS, or oxidative phosphorylation inhibitors are known causes of autophagy activation in order to avoid a lethal increase of ROS and proapoptotic factors [32]. Such an important keystone to maintain homeostasis is regulated by intricate pathways that are described below.

### Mitophagy Machinery: A Mitochondrial-Selective Autophagy

First, autophagy must be regarded as a highly dynamic process along time and space, where a great diversity of proteins is involved to ensure its regulation. Two classical autophagy master keys are the mammalian targets of rapamycin complex 1 (mTORC1) and AMP-activated protein kinase (AMPK). The first one is in charge of the inhibition of this pathway through the phosphorylation of Unc-51 like autophagy activating kinase 1 (ULK1) when autophagy is not needed (e.g., in abundant nutrient-rich situations). Alternatively, AMPK also phosphorylates ULK1 resulting in its activation and triggering autophagy. Thus, a balance in both kinase activities is needed to achieve correct levels of this catabolic pathway [33]. Autophagy begins with the formation of the phagophore, a lipid membrane that selectively elongates over the cargo that must be degraded, whether mitochondrion or another kind of target [34]. In any case, cargo is marked with microtubule-associated proteins 1A/1B light chain 3B (LC3) in order to discriminate those to be degraded. After that, the phagophore becomes a double-membrane vesicle called an autophagosome, probably through the fission of the pre-autophagosomal membrane [28]. The double-membrane vesicle is transported along the axon to the soma, where huge populations of lysosomes are found. Finally, the autophagosome merges with the lysosome, generating the autolysosome, where the cargo is degraded by hydrolytic enzymes [35].

**Figure 2:**
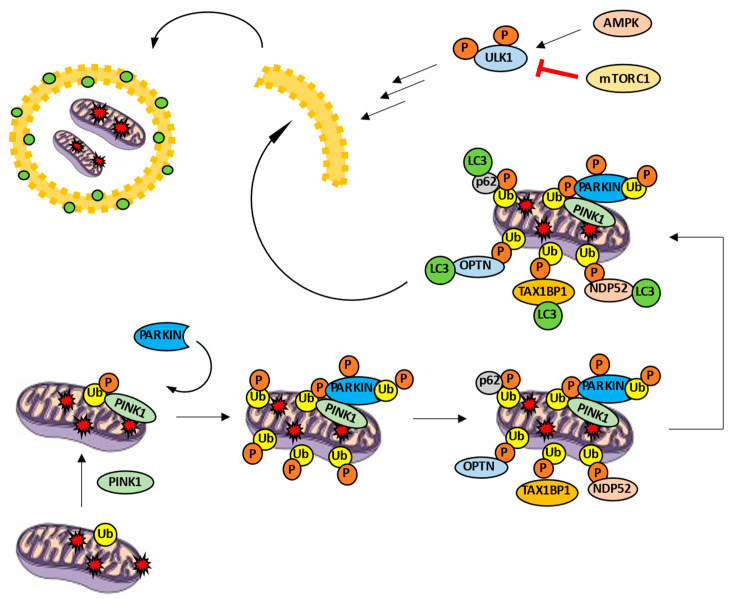
PINK1/PARKIN pathway in mitophagy activity. PINK1 is accumulated in the mitochondrial surface and phosphorylates ubiquitin, recruiting PARKIN ubiquitin ligase. PARKIN is phosphorylated and increases the number of ubiquitins. Autophagy receptors such as *OPTN*, p62, NDP52 or TAX1BP1 link to the phospho-ubiquitin and LC3, promoting the formation of the autophagosome around the cargo.

Mitophagy, as a selective variant of autophagy, is characterized by molecular mechanisms that allow selective degradation of mitochondria. The most studied mechanism is the PINK1/PARKIN pathway. PTEN-induced kinase 1 (PINK1) is a cytoplasmatic kinase able to phosphorylate different substrates, including ubiquitin and PARKIN. On the other hand, PARKIN is a E3 ubiquitin-ligase [36,37]. In the case of a healthy mitochondrion, PINK1 is imported into the mitochondrion through the translocases of the outer and inner membrane (TOM and TIM, respectively) complexes. Afterwards, PINK1 is degraded inside the organelle [32]. However, when the mitochondrion is damaged, TOM and TIM complexes are not able to import PINK1, which is the kinase accumulated on the mitochondrial surface. PINK1 phosphorylates ubiquitin, turns it into an allosteric modulator of PARKIN and prompts its recruitment [38]. PINK1 is also able to phosphorylate outer membrane proteins such as mitofusin 2 (MFN2), facilitating the recruitment of the ubiquitin-ligase as well [39]. Subsequently, PINK1 phosphorylates PARKIN, producing a conformational change and discovering its ubiquitin-ligase activity on various proteins such as MFN1, MFN2, voltage-dependent anion channel 1 (VDAC1), and MIRO1 [32,38]. Therefore, ubiquitin is both a substrate and a PARKIN modulator. Once the mitochondrion is polyubiquitinated, autophagy receptors such us optineurin (*OPTN*), p62, nuclear dot protein 52 (NDP52), TAX1 binding protein 1 (TAX1BP1), and neighbor of BRCA1 gene 1 (NBR1) facilitate the formation of autophagosome around the defective mitochondrion [40,41]. These receptors have at least two domains: one responsible for recognizing ubiquitin and another able to link LC3 (LC3-interaction region, LIR motif). This way, only ubiquitinated mitochondria are marked LC3-positive and, hence, are degraded (Figure 2).

Interestingly, PINK1 is able to recruit *OPTN* and NDP52 by means of phospho-ubiquitin without PARKIN activity [42]. Moreover, knockout models point to only *OPTN* and NDP52 as strictly necessary autophagy receptors to trigger mitophagy through PINK/PARKIN pathway and overshadow the rest of receptors [42]. Besides, *OPTN* and NDP52 receptors are able to recruit ULK1 by themselves and promote phagophore formation [40,42]. It is worth nothing that the role of p62 as a possible dispensable receptor keeps generating controversy [41]. All the above leads us to think that PARKIN functions as a PINK1 modulator: PARKIN facilitates an increase of ubiquitins on the outer mitochondrial membrane. Then, these ubiquitins can be phosphorylated by PINK1 in order to recruit a greater number of autophagy receptors. Without this positive feedback mechanism, mitophagy levels would never be enough due to the low constitutive amount of ubiquitin in the mitochondrion. This explains, at least in part, the mild phenotype observed in PARKIN^−/−^ models [43].

The paragraphs above describe the PINK1/PARKIN-dependent mitophagy. However, other pathways to trigger mitophagy have been reported in the last years. First of all, LC3 is considered a canonical ATG8 yeast homologue in humans and is extremely useful in autophagy studies. However, other ATG8 homologues have been reported in mammals with implications for autophagy. Indeed, the ATG8 homologue gamma-aminobutyric acid receptor-associated protein-like 1 (GABARAPL1) is highly expressed in motoneurons [44]. On the other hand, cardiolipin can interact with LC3 without PINK1/PARKIN activity. Under mitochondrial damage conditions, inner mitochondrial membrane lipid cardiolipin can flow to the outer membrane and initiate mitophagy [45]. Moreover, several proteins on the outer mitochondrial membrane contain LIR motifs, which ensure a direct interaction between defective mitochondria and LC3. Some examples are autophagy and BECLIN 1 regulator 1 (AMBRA1) and FUN14 domain containing protein 1 (FUNDC1) [32,45,46]. Finally, there are other ubiquitin-ligase proteins like PARKIN that are capable of participating in mitophagy such as the mitochondrial ubiquitin-ligase activator of NF-kB (MULAN) and the membrane-associated ring finger 5 ubiquitin-ligase (MARCH5) [45]. The fact that PINK1/PARKIN-dependent pathway is not responsible for all mitophagy activity brings new pieces to the unsolved puzzle of mitochondrial quality control. Future investigations may declare these alternative pathways as interesting targets in mitophagy-altered diseases.

## 4. Mitophagy and ALS

As we exposed before, mitochondrial quality control is tightly regulated through the orchestrated action of several pathways. Taking into consideration that maintaining a healthy pool of mitochondria in neurons is something critical in order to ensure their functionality, mitophagy mechanisms must work properly to guarantee a reduced level of defective mitochondria. Nevertheless, the large number of proteins involved in mitophagy provides several hot points where diseases can be triggered. In the case of ALS, many defects in mitochondria degradation have been documented in the last years. The role of PINK1/PARKIN has been extensively studied in multiple neurodegenerative diseases and there is a certain consensus about its neuroprotective action. However, its role in ALS still produces controversy. TDP-43 is responsible, under physiological conditions, for regulating the transcription of many mRNAs, including PARKIN mRNA. The spinal cords of sALS patients with TDP-43 accumulation has been found to show a decrease in PARKIN levels [47]. *SOD1*^G93A^ mice models exhibit a highly stimulated mitophagy at early stages of the disease despite a reduction in spinal cord PARKIN levels [48]. When PARKIN is knocked out in this model, mice show an increase in survival rate. The authors argue that PARKIN could have a short-term neuroprotective effect, but a long continued mitophagy activity would lead to defective mitochondrial machinery and be detrimental in the long term. In flies expressing both *FUS* and *FUS*^P525L^, PINK1 and PARKIN levels are increased, while their reduction by siRNA improves neurodegenerative phenotypes caused by *FUS* [49]. Nonetheless, a recent study on flies shows the reversal of locomotive defects caused by *FUS* through PARKIN expression [50].

Autophagy receptor defects have been found in several ALS types. There are about forty different ALS-related *OPTN* mutations, which contribute to 1% of fALS cases, although they can also be observed in sALS patients [3]. For example, ALS-related mutations such as E478G and Q398X prevent *OPTN*-ubiquitin binding and impair mitophagy [40,51]. Other functions have been associated with this receptor; since *OPTN* is able to bind LC3 and myosin VI, it is able to regulate autophagosome flux through the neuron [52]. Moreover, E478G and Q398W mutations inhibit the fusion of autophagosomes with lysosomes in NSC-34 cells, a widely used motoneuron model [40]. Recently, an interesting link between *SOD1* aggregates and *OPTN* was also reported: *SOD1* aggregates retain *OPTN* and interfere with the mitophagy machinery [53]. On the other hand, mutations in *SQSTM1* (the gene that encodes for p62) embrace 1% of fALS cases [3]. Its role in protein degradation is widely known and, in the same way as LC3, p62 can be used as a marker in autophagy defect studies. In addition, p62 can be found in protein aggregates of some ALS patients and its depletion has been shown to shorten life expectancy in *SOD1*^H46R^ and *SOD1*^G93A^ transgenic models [54]. In the last years, the relevance of the TANK-binding kinase 1 (*TBK1*) as a mitophagy modulator has been highlighted. This serine/threonine kinase is able to phosphorylate p62, NDP52, and *OPTN* [41], increasing its ability to link ubiquitin and LC3. Autophagic receptors are capable of binding ubiquitin, resulting in low mitophagy activity. This binding causes *TBK1* activation and the consequent phosphorylation of autophagic receptors, increasing their activity [55]. Besides, *TBK1* induces *TBK1*-*OPTN* retention on the surface of damaged mitochondria. More than eighty ALS-related *TBK1* mutations have been reported [41]. For example, p.690-713 deletion of *TBK1* at C-terminal impairs its binding with *OPTN* and compromises mitophagy [56]. *TBK1* may also play a regulatory role in the onset of mitophagy since C9orf78 is able to promote mitophagy through the interaction with ULK1 complex due to its binding with the Smith–Magenis syndrome chromosome region candidate 8 (SMCR8), a guanine nucleotide exchange protein. C9orf78 is activated by *TBK1* through an SMCR8 *TBK1*-dependent phosphorylation [57]. *TBK1* may also alter later processes of mitophagy such as the transport of autophagosomes. This can be explained due to the role of *TBK1* in the regulation of microtubule polarization during mitosis, as well as the regulation of cytoplasmatic levels of dynein [41].

As we exposed before, one of the strongest indicators correlating mitophagy and ALS is the fact that several ALS-related mutations lead to the misfunction of proteins such as *OPTN*, p62, or *TBK1* and, therefore, an inefficient mitophagy. However, the majority of sALS patients do not present these kinds of mutations, raising the following question: is mitophagy modulation an interesting topic for all the ALS patients or only for those who present mitophagy mutations? Mitochondria can be considered as a convergence point in ALS pathology. Mitochondria defects can be associated with most of the pathways involved in ALS, as the accumulation of dysfunctional mitochondria is related to inadequate ATP generation, high ROS levels, altered Ca^2+^ buffering, and cell death. Thus, ensuring a healthy pool of mitochondria is crucial to improve ALS pathology, which includes correct mitophagy levels, regardless of whether patients present mitophagy mutations or not. On another note, TDP-43 has been reported to alter several mitochondrial pathways such as mitochondrial dynamics, mitochondrial trafficking, and energetic metabolism [58]. Recently, it has also been documented that TDP-43^M337V^ mice show increased levels of TDP-43 in mitochondria with corresponding mitochondrial damage and cell death. Surprisingly, blocking the link between TDP-43 and mitochondrion is enough to alleviate the neuronal loss and mitochondrial dysfunction [59]. Thus, preventing TDP-43-mitochondrion interaction and degrading these defective mitochondria may be a promising approach in order to treat ALS. Since TDP-43 aggregates are found in the vast majority of patients, ensuring an adequate mitophagy activity in order to maintain a healthy pool of mitochondria is a promising opportunity for ALS treatment.

### 4.1. Mitophagy and Neuroinflammation in ALS

Mitophagy and neuroinflammation are interestingly overlapped because of the pleiotropic character of the genes involved. Neuroinflammation is usually related to the reaction of glial cells (astrocytes, microglia, and oligodendrocytes) and circulating immune cells (such as lymphocytes) to infection, injury, or degeneration. Therefore, long-term overproduction of inflammatory mediators eventually produces cell death. A wide consensus has been achieved on the correlation between neuroinflammation and neurodegenerative diseases. The relation between the *OPTN* autophagy receptor and neuroinflammation is increasingly striking. Knockdown [60] and knockout [61] models show an *OPTN* inhibitory role on nuclear factor-kappa B (NF-kB) in vitro. Interestingly, ALS-related mutations Q398X and E378G do not exhibit this kind of activity [40]. On the other hand, *TBK1* is known to regulate neuroinflammation through type I interferon (IFN). This kinase acts downstream of toll-like receptor 3 (TLR3), which is implicated in the immune response, as *TBK1* is an activator of interferon regulatory factor 3 (IRF3) and results in an increase of IFNα, β, and γ [62]. Since the treatment of ALS patients with IFNα and β leads to strong inflammation [63,64], *TBK1* activity is expected to increase neuroinflammation and have a negative role in ALS. In contrast, *TBK1* could have a protective role in these glial cells. Circulating T-cells are thought to stabilize microglia and reduce proinflammatory cytokines levels. *TBK1*^-/-^ T cells show a reduced migration to the central nervous system (CNS), which could reduce the number of T cells infiltrated in the CNS and result in increased damage from ALS [65]. Besides, it has been documented that *TBK1* is a natural endogenous inhibitor of receptor-like protein kinase 1 (RLPK1), which is highly related to apoptosis and inflammation events [66]. The pleiotropic nature of *TBK1* should be carefully considered before declaring any statement. To sum up, since many alterations related to neurodegeneration can lead to mitophagy defects and neuroinflammation, the overlapping field between both rises as an interesting topic of research.

### 4.2. Mitophagy as Target

In the recent years, a growing number of articles have been reported to link ALS, autophagy, and mitophagy. An increase in autophagosomes and autolysosomes highly rich in mitochondria in the spinal cords of ALS patients have been described [67]. The ablation of genes involved in autophagy is enough to trigger neurodegeneration in murine models [68,69] and, as it is described above, multiple ALS-related genes are directly involved in mitophagy pathways such as *OPTN*, *TBK1*, or *SQSTM1*. Therefore, many autophagy-stimulating drugs have been probed in ALS models and studied to determine their pharmacological action. Several molecules are able to reduce TDP-43 aggregates by means of autophagy stimulation, some examples are rapamycin or trehalose. However, the effects of autophagy modulators rely heavily upon the ALS model used [70]. Verapamil has been shown to delay the onset of the disease, increase life expectancy, regain the functionality of affected motor neurons, and reduce the aggregation of *SOD1* by increasing autophagy in the *SOD1*^G93A^ model [71]. At the same time, its ability to enhance autophagosome–lysosome fusion is well known [72]. Long-term rilmenidine use, a common anti-hypertensive, promotes mTOR-independent autophagy in the spinal cord of *SOD1*^G93A^, hardly reducing the onset of hind-limb paralysis and producing depletion of mitochondrial levels; this is accompanied by an accelerated motoneuron degeneration [73]. Autophagic stimulation of clemastine shows beneficial effects only in the short term, and presents no effect on survival or disease progression in the long term [74]. Rapamycin shows null or negative effects on *SOD1* models, but beneficial effects in a TDP-43 model [70]. This issue is more raveled with respect to trehalose, where contradictory data can be found. In 2013, trehalose was reported to show beneficial effects in the disease progression of a *SOD1*^G86R^ mouse model [75], in the same way that *SOD1*^G93A^ mouse was reported [76]. Two years later, by contrast, trehalose was reported to perform beneficial roles only in early stages in the *SOD1*^G93A^ model [77]. Taking all these data into consideration, the huge number of ALS models, and the diversity at the time of administrating the drug, making absolute statements about this issue is risky. The paragraphs below try to bring light to the role of mitochondria in this brainteaser.

#### 4.2.1. Mitophagy Is a Highly (Temporal) Dynamic Puzzle

**Figure 3:**
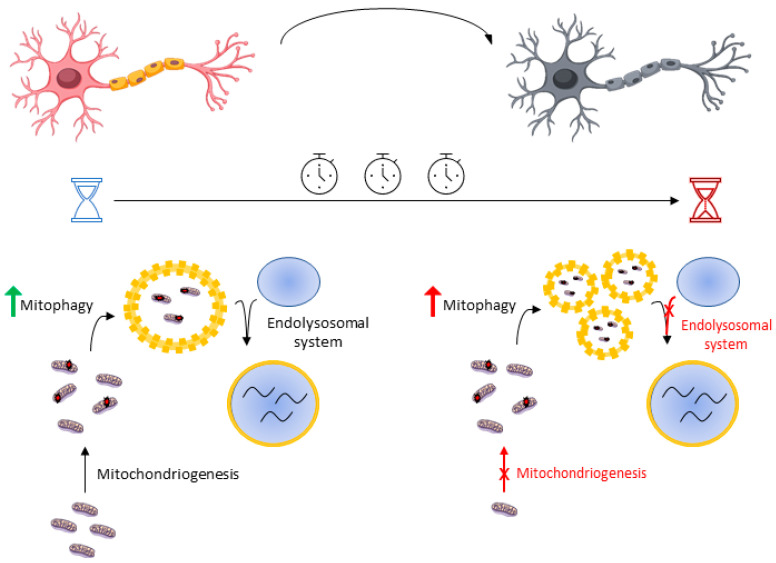
Mitophagy induction shows different effects at early and late stages. Boosting mitophagy may be beneficial in the short term as mitochondriogenesis and the endolysosomal system work properly. However, this strategy may be detrimental in the long term due to progressive defects in both mitochondriogenesis and the endolysosomal system, resulting in the accumulation of nonfunctional vacuolated organelles.

Mitophagy is a fundamental mechanism in the maintenance of homeostasis, so the elimination of defective mitochondria is critical for the proper functioning of the neuron. However, an excess of mitophagy activity can result in the reduction of functional mitochondria, so this strategy could not only be inefficient but even harmful. Mitophagy activity must be in balance with the mitochondrial biogenesis or mitochondriogenesis, a set of mechanisms aimed at increasing mitochondrial machinery. Peroxisome proliferator-activated receptor gamma coactivator 1-alpha (PGC-1α) is one of the major transcription factors that regulate mitochondriogenesis. Although low levels of PGC-1α have been detected in animal models and ALS patients [78], mRNA PGC1-α was progressively increased until 60 days of life in *SOD1*^G93A^ mouse and decreased from 90 days until death [48]. Keeping this in mind, one possible hypothesis that may explain this is a progressive imbalance between mitophagy and mitochondriogenesis. At early stages of the disease, mitophagy induction may prove beneficial as mitochondriogenesis provides new and healthy mitochondria, maintaining appropriate levels of functional mitochondria. In contrast, mitophagy induction at late stages would result in a deficit of mitochondrial machinery due to an inefficient biogenesis. On the other hand, the *SOD1*^G93A^ transgenic model shows a markedly progressive deficiency of the endolysosomal system activity, that is, the set of mechanisms aimed to transport autophagosomes and merge them with the lysosomes [79]. Time-lapse studies have shown that the temporal dynamics of neuronal mitophagy are very slow, in which lysosomal fusion and acidification are the rate-limiting stages [80]. Indeed, several ALS-related mutations in genes such as *C9orf72*, *ALS2*, or *CHMPB2* are involved in autophagosome maturation defects and their fusion with the lysosomes [70]. All of this defines a hypothetical model where mitophagy induction may be detrimental at late stages of neurodegeneration due to an accumulation of nonfunctional vacuolated organelles and the inability of the neuron to provide new healthy mitochondria (Figure 3). According to the information exposed before, enhancing lysosomal function could be an interesting strategy over forcing general autophagy. This shows the highly dynamic nature of this kind of pathway and the need to display not only a strong autophagy or mitophagy, but a time-coordinated mitochondrial quality control. It is therefore not surprising that autophagy inductors, such as lithium [81] or resveratrol [82], also increase mitochondrial biogenesis. It is of note that a sole increase in mitochondriogenesis rate without autophagy stimulation cannot extend survival in ALS mouse models despite showing improvements in muscle activity [83]. Ultimately, better knowledge about autophagy machinery based on disease stage would provide clues about which part of mitochondrial quality control we need to enhance.

#### 4.2.2. Mitophagy Is a Highly (Neuron-Type) Dynamic Puzzle

In 2006 and in parallel, CNS-selective deletion of autophagy models were generated in order to study the role of autophagy in neurodegenerative diseases. As is well known, deletion of *ATG5* [68] or *ATG7* [69] (both essential genes for autophagy activity) in the CNS leads to neurodegenerative phenotypes, so the spotlight was put on autophagy machinery in the race to discover an effective treatment for ALS. Although ALS was initially considered as a pure neurodegenerative disease, subsequent reports attributed a muscle-disease nature, since motoneuron degeneration in ALS models is preceded by denervation of the neuromuscular junction (NMJ) [84,85]. Therefore, the NMJ and motoneuron received the attention they deserved. Recently, different roles in autophagy at early and late stages of ALS were assigned due to a noncell autonomous progression of the disease in [86]. *ATG7* elimination, specifically in motoneurons (*ATG7* cKO) in *SOD1*, showed an impaired autophagy activity only in these kinds of neurons. Surprisingly, mice did not present neurodegenerative phenotypes and their motoneurons remained viable. Nevertheless, mice displayed structural and functional defects in the NMJ of some of these autophagy-truncated motoneurons, including the denervation of the tibialis anterior, which is mostly innerved by fast motoneurons. Additionally, they documented the disease progression of the *SOD1*^G93A^ mouse model. They reported that only vulnerable and first-to-die motoneurons display round body aggregates that are GABARAPL1-positive at early stages, while nonvulnerable motoneurons present skeinlike aggregates that are GABARAPL1-negative at late stages. Typically, fast motoneurons die first at early stages of ALS, while slow motoneurons are more resistant and remain functional. The authors argue that fast motoneurons are able to activate autophagy machinery according to the fact that they are GABARAPL1-positive. Consequently, slow and resistant motoneurons do not recruit autophagy machinery at the same level as fast ones do. Intelligently, the authors crossed *SOD1*^G93A^ and *ATG7* cKO mouse models in order to explore the role of autophagy in this intriguing puzzle. As expected, fast motoneurons were affected early, resulting in the accelerated denervation of the tibialis anterior muscle. The soleus, innervated principally by slow motoneurons, remained innervated even late in the disease. The onset of tremor in this mouse model was reported earlier than in *SOD1*^G93A^ due to the misfunction of these fast motoneurons. However, authors reported an extended lifespan of mice and a delay in disease progression accompanied by a reduction in glial inflammation. That is, detrimental effects of autophagy activity in motoneurons could be projected to the glia cells in the CNS in a “dying-back” communication. Several highlights can be extracted from these results: (i) selective autophagy impairment in motoneurons is not enough to trigger neurodegeneration; (ii) autophagy may be beneficial in the short term but detrimental in late stages of the disease; (iii) denervation in vulnerable motoneurons may course in a cell-autonomous way in *SOD1*^G93A^ when autophagy is impaired but iv) disease progression is probably mediated by noncell autonomous mechanism—glia cells are an interesting point to be considered.

**Figure 4:**
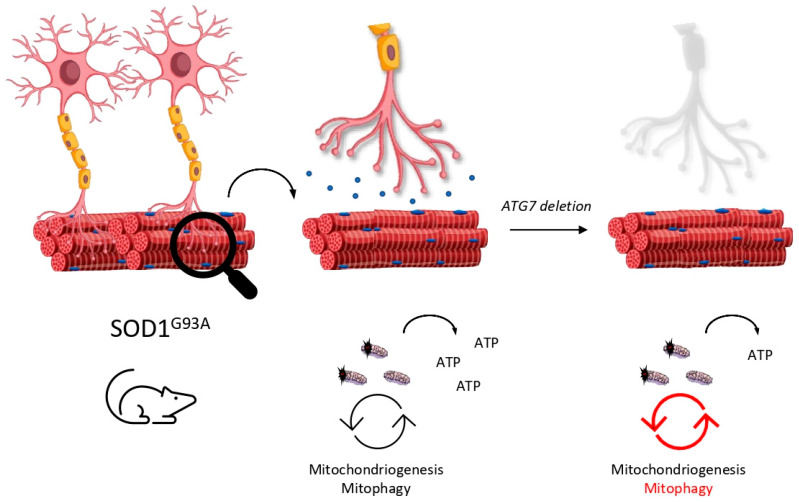
Mitophagy is essential to maintain the neuromuscular junction (NMJ). Inefficient renewal of mitochondria due to the selective deletion of *ATG7* in motoneurons may lead to low le-vels of ATP and make them prone to denervation.

According to this, why are fast motoneurons more vulnerable than the slow ones? Fast motoneurons innerve large muscles and can be crudely divided into fatigable (FF) or resistant (FR) [87]. FF motoneurons, like those that innerve tibialis muscle, have high peak needs of ATP [88], thus, they need to maintain their mitochondria pool in good conditions to ensure the correct level of ATP. ALS patients [24] show impaired production of ATP and *SOD1*^G93A^ present a low ATP/AMP ratio due to a deficient oxidative phosphorylation metabolism at early stages of the disease [89]. Recently, mitochondria located in the synapsis of spinal cord motoneurons were reported to suffer an enhancement of glucose catabolism in order to supply the inefficient production of ATP at the presymptomatic stage, while this only occurs later in the cortex motoneuron and perisynaptic glia cells [90]. An interesting hypothesis is that *SOD1*^G93A^ models display deficient mitochondrial function and inadequate ATP levels. Knowing that FF motoneurons usually present a low number of mitochondria and are rapidly fatigable [91], the poor energy supply affects these motoneurons drastically due to their high metabolic demand. When autophagy is impaired, FF motoneurons are not able to degrade these defective mitochondria and provide themselves with new inputs to cellular metabolism, ending up with denervation in the short term (Figure 4). However, if autophagy is stimulated, misfunction of the NMJ is delayed, but motoneuron accumulates nonfunctional vacuolated organelles that cause earlier cell death at latest stages, as we exposed before. Accordingly, PINK1/PARKIN double KO mice are prone to accumulate defective mitochondria in the NMJ and, subsequently, cause denervation [92]. It is of note that FF motoneurons display a huge glycolytic metabolism as an energy source, thus, impaired mitochondrial ATP production may not be the unique reason for why this kind of neuron is affected first [87]. Instead, a set of features must converge in FF motoneurons to explain this selectivity. The short latency period between action potentials or the earlier endoplasmic reticulum stress are two of the possible causes [93]. Interestingly, vulnerable motoneurons display lower Ca^2+^ buffering compared with other kinds of neurons, which allows fast recovery times during physical exercise [94]. As a result, mitochondrion acts as an even more critical Ca^2+^ storage in these neurons. Since FF motoneurons show impaired Ca^2+^ handling at presymptomatic stages [95] and dispose of a low number of mitochondria, the accumulation of defective mitochondria may lead to a dysregulation in Ca^2+^ levels and consequent cell damage. Therefore, a correct balance between mitophagy and mitochondriogenesis is needed to maintain a healthy pool of these organelles and ensure an adequate intracellular concentration of Ca^2+^.

#### 4.2.3. Mitophagy Is a Highly (Cell-Type) Dynamic Puzzle

**Figure 5:**
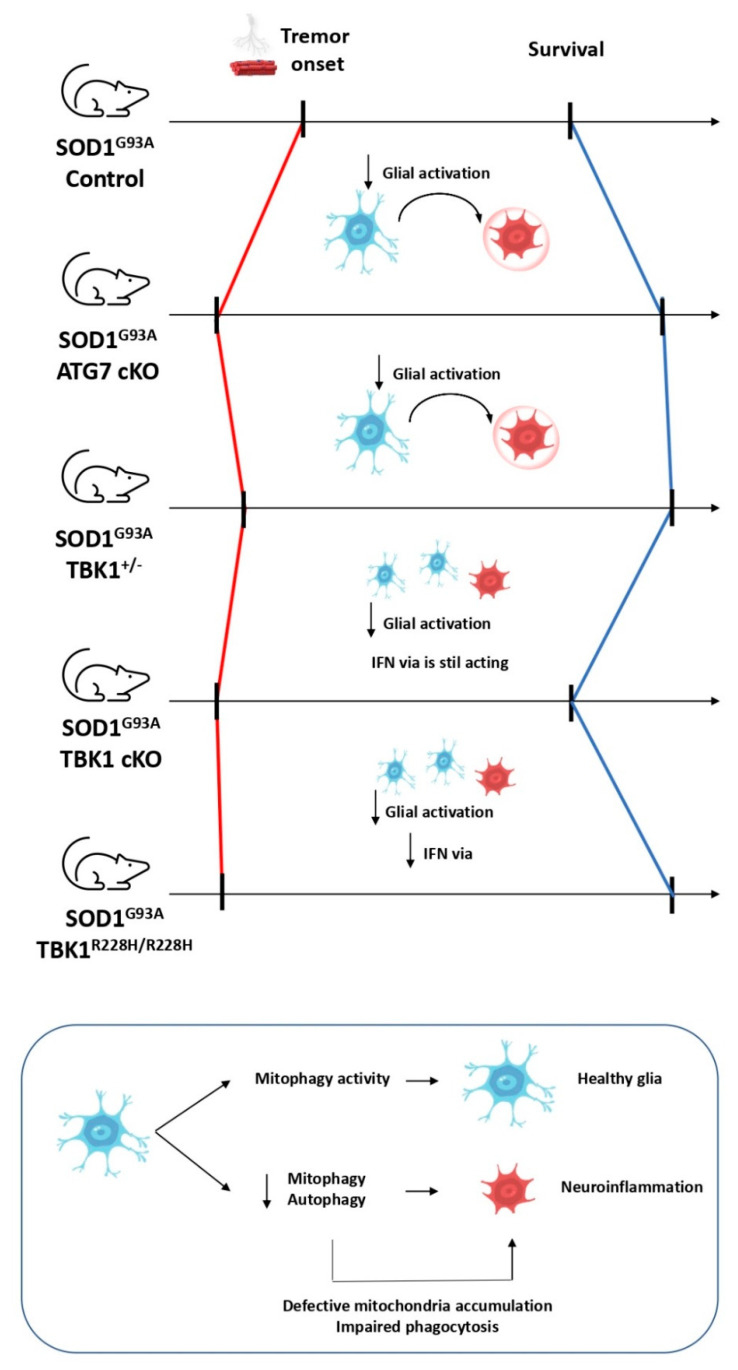
ALS progression presents a noncell autonomous nature. Selective deletion of autophagy genes in motoneurons causes earlier denervation in these cells. However, the *SOD1* mouse model presents an extended survival, probably due to the role of glia cells in neuroinflammation.

Neuroinflammation by glial cells is recognized as an important disease modulator. Although there is still controversy about the relationship between them, autophagy stimulation is believed to reduce neuroinflammation in microglia by restricting inflammasome activation and preventing elevated ROS levels through mitophagy activity, among other processes [96]. Specifically, glial cells are broadly recognized as important modulators of ALS, which can be observed in the fact that transplanted wildtype microglia delays disease progression in ALS models [97] and the fact that selective deletion of mutant *SOD1* in astrocytes affects disease progression but not its onset [98]. In the same way, selective deletion of mutant *SOD1* in microglia clearly influences late stages but not early stages [99]. The work we discussed above provides the opportunity to study the course of ALS when autophagy is only limited to motoneurons, which is not possible with the use of autophagy modulators such as trehalose or rapamycin. The alleviation of neuroinflammation in the *ATG7* cKO/*SOD1*^G93A^ mouse model is probably due to the reduction of those vacuolated organelles that would stress neurons in the long term, but the fact that autophagy is not impaired in glial cells cannot be ignored. Therefore, could we suppose that the coelimination of autophagy activity by gene deletion in microglia and motoneurons would counteract this alleviation and induce neuroinflammation? It is a hard-to-solve question if we take into consideration that mutant *SOD1* can sequester *OPTN* and impair mitophagy; thus, using the mutant *SOD1* model implies the risk that mitophagy can be basally interfered in the control group [53]. In 2019, a heterozygous *TBK1*^+/−^/*SOD1*^G93A^ mouse model was designed and studied [100]. As we exposed before, *TBK1* is a key kinase that has the critical role of ensuring correct levels of mitophagy and is involved in inflammatory responses via IFN. Accordingly, *TBK1*^+/−^/*SOD1*^G93A^ mice showed increased denervation in pretibial muscles and the onset of the tremor was accelerated. Moreover, at later stages, neuroinflammation was reduced, disease progression was delayed, and survival was extended. Authors argue that the deleterious autophagy/mitophagy inhibition effects can be observed at 50 days, but the beneficial impact of reducing microglia activation is overcome at 120 days. Due to the pleiotropic nature of *TBK1*, these data are far from revealing whether the reduction in microgliosis may correspond to either a reduction of autophagy/mitophagy activity or a decrease in neuroinflammation. Conveniently, motoneuron-selective *TBK1* deletion (*TBK1* cKO) was recently designed in 2020 [101]. Thus, several mouse models have been produced such as *SOD1*^G93A^, *TBK1*^R228H/R228H^/*SOD1*^G93A^ (where mutated *TBK1* shows no activity in any cell type), *TBK1*^R228H/+^/*SOD1*^G93A^ (where *TBK1* acts in a heterozygous way), and *TBK1* cKO/*SOD1*^G93A^ (where *TBK1* is deleted only in motoneurons). According to the work discussed before, *TBK1*^R228H/R228H^/*SOD1*^G93A^ and *TBK1*^R228H/+^/*SOD1*^G93A^ mice present more denervation in early stages but extended survival rates compared to *SOD1*^G93A^ mice. However, *TBK1* cKO/*SOD1*^G93A^ mice present more denervation and earlier onset of the disease but without an effect on the mice survival. This indicates that earlier denervation can be observed in a cell autonomous way when either *ATG7* or *TBK1* are deleted in motoneurons, but the disease progression is completed, at least in part, in a noncell autonomous way, as only noncell selective impairment of *TBK1* (*TBK1*^R228H/R228H^/*SOD1*^G93A^) extends survival. Moreover, this prolonged survival in *TBK1*^R228H/R228H^/*SOD1*^G93A^ is accompanied by a lower induction of a subset of interferon stimulated genes (ISGs) in glial cells, specifically in astrocytes and microglia. Therefore, neuroinflammation produced by glial cells is linked to disease progression and may be responsible for its extended lifespan (Figure 5). All the data are complemented by the fact that (i) selective-mononuclear phagocytic system deletion of *ATG7* (which includes microglia) causes intestinal adhesion of macrophages, which is strongly related with inflammatory events [102], and (ii) mitophagy activity in microglia reduces neuroinflammation in Alzheimer’s disease [103]. Hence, autophagy/mitophagy in microglia may be important to ensure low levels of neuroinflammation.

In 2020, an alternative mitochondrial quality control was reported. It was observed that the neuroblastoma cell line SH-SY5Y, among others, releases mitochondria to extracellular space [104]. Furthermore, mitochondrial damage induced by small molecules, such as rotenone, carbonyl cyanide m-chlorophenyl hydrazone (CCCP), or mitophagy-deficient models like *PARKIN* mutations, increase the mitochondrial release. Extracellular mitochondria must be eliminated in order to avoid neuroinflammation, since several mitochondrial components such as cytochrome C, mitochondrial transcription factor A (TFAM), or cardiolipin can act as damage-associated molecular patterns (DAMPs) and lead to inflammatory events [105]. In parallel, a Parkinson’s disease model was documented in which defective mitochondria were released in spheroids when the dopaminergic synapsis was degenerated. Surprisingly, these mitochondria were completely degraded not in the extracellular space, but in concomitant astrocytes, where the final steps of mitophagy are performed [106]. Therefore, glial cells (at least astrocytes) show the ability to degrade these released mitochondria through a process termed as transmitophagy, in which mitochondrial degradation in neurons can be mediated by intercellular pathways. This mitochondrial delivery from the affected cell to the glia (and vice versa) has been documented not only in neurons, but also in many other cell types in the last years [107]. However, the fact that impaired mitophagy promotes this mitochondrial release opens new research fields. Phagocytosis-mediated elimination of extracellular mitochondria would end up with the delivered organelles to lysosomes, so it is not surprising that autophagy and phagocytosis share common molecular machinery as they are in charge of the elimination of intracellular and extracellular components, respectively. Indeed, a kind of endocytic pathway has been described as the LC3-associated phagocytosis (LAP), characterized by the recruitment of LC3 to the phagosome membranes involved in the phagocytosis, responsible for the degradation of several types of cargo, such as pathogens or cell debris [108]. In this context, a paradigmatic case was reported recently about microglia-selective deletion of *ATG7* in a multiple sclerosis model: defective autophagy-related phagocytosis in microglia impairs myelin debris elimination. The accumulation of these remains of myelin leads to neuroinflammation and the inability to remyelinate neurons, due to the lack of *ATG7* [109]. Curiously, trehalose is able to reverse the neurodegenerative phenotype in the aged mouse model because of its autophagy stimulation activity. These data raise the possibility that autophagy machinery is not only needed in microglia to avoid neuroinflammation, but is also necessary to degrade those extracellular mitochondria by glial cells (Figure 5). However, experiments in ALS models and other neurodegenerative diseases are needed to confirm this.

## 5. What Is on the Immediate Horizon?

As a summary, this review attempts to provide an approach to investigate the role that mitochondria and mitophagy may display in ALS models. Firstly, we have provided a feasible explanation of why some autophagy and mitophagy enhancers only present beneficial effects in the short term, remaining aware of the fact that mitochondria quality control is a multisided puzzle in which mitophagy must be in balance with other pathways such as mitochondriogenesis and lysosomal function, among others. Secondly, we have remarked the fact that selective degradation of *ATG7* in motoneurons is not enough to trigger neurodegeneration. Moreover, depending on the nature of the motoneuron, they can be more- or less-affected by the autophagy impairment, underscoring the point that those first-to-die motoneurons are more susceptible to energy defects, so inadequate ATP production due to defective mitochondria may be behind this selectivity. Thirdly, we have highlighted how glial activity is an important disease modulator and the need of adequate levels of autophagy/mitophagy to avoid neuroinflammation.

However, most of the evidence shown has been obtained from the *SOD1*^G93A^ mouse model. This transgenic animal was created in 1994 and has been widely used to study ALS since. *SOD1*^G93A^ has allowed researchers to understand fundamental parts of ALS pathology like mitochondrial defects, deficit axonal transport, glutamate toxicity, and others, but routine use of other models is needed to dive deeper into ALS [110]. In fact, most clinical efforts based on this model usually tend to fail, probably because the great majority of ALS patients are sporadic, and *SOD1*-mutations are present only in 20% of fALS patients. We strongly believe that, due to the complex pathology of ALS, more than one model is required to reflect this complexity. Since more than 90% of the patients present TDP-43 aggregates and mutations in *C9orf72* are linked to 40% of fALS cases, these transgenic models can offer new perspectives in the search of effective treatments. We cannot discard that remarkable differences may appear in studies with the different models. For example, the hemizygous *TBK1* TDP-43 mouse model (TDP-43^G298S^/*TBK1*^+/−^) presents more NMJ denervation and slight motor function impairments compared to TDP-43^G298S^ without changing signals in autophagy and glial activity [111]. This could mean that biological effects may be in agreement, but mechanistic differences are behind common phenotypes. Collecting and harmonizing results from the different models is a pending task that we must complete if we really intend to understand the role of these processes in ALS pathology. On the other hand, most ALS studies usually focus on spinal pathology, as bulbar-onset ALS is one of the most devastating variants of the disease characterized by a rapid disease progression, an even shorter life expectancy (less than two years), and cognitive/language impairment [112]. To our knowledge, very few studies are dedicated to deciphering the role of mitochondria in different variants of ALS. One attempt to clarify this was recently reported, where authors evaluated both bulbar and spinal disease in *SOD1*^G93A^ mice through the study of the citrate synthase (CS), among other markers. CS is altered in the extensor digitorum longus (mostly innervated by fast motoneurons) and soleus muscle (mostly innervated by slow motoneurons), while CS remains unaltered in the tongue muscle, and is typically affected in bulbar ALS [113]. In the same way that mitochondria may play a feasible role in the selectivity between fast and slow motoneurons, further studies are needed to clarify if differences between bulbar- and spinal-onset ALS could be related to unexplored roles of mitochondria.

Furthermore, these kinds of pathways have a very dynamic nature. One possibility is that autophagy enhancers are not beneficial during the whole course of the disease. It is possible that the sole autophagy enhancement could be positive only in early stages of the disease, while during later stages, autophagy inducement should be accompanied by drugs that promote mitochondriogenesis and lysosome function, ensuring a correct mitochondrial quality. Viewed in these terms, multitarget drugs are an interesting perspective to prevent autophagy-related neurodegenerative diseases, including ALS. Finally, cell-type-selective deletion of autophagy genes has brought new perspectives about the influence of autophagy in ALS. Moreover, it has remarked the role of glial cells in the progression of the disease and the hypothesis of the noncell autonomous course. Similarly, the search for new proteins that selectively modulate autophagy events in either glial cells or motoneurons would offer the opportunity to design selective autophagic modulators and expand the field of ALS treatment. An example may be P2X purinoceptor 7 (P2X7), a ligand-gated ion channel receptor involved in autophagy events [114]. P2X7 is ubiquitously expressed in humans but particularly in myeloid cells, which include microglia, and it is involved in neuroinflammation through extracellular ATP activity, resulting in a neuron-microglia communication mechanism. Short time stimulation of this receptor enhances autophagy in microglia and reduces neuroinflammation, while prolonged stimulation causes accumulation of p62-positive aggregates. Remarkably, prolonged treatment with A-804598, the antagonist of this receptor, reduces p62-positive aggregates in the spinal cord of *SOD1*^G93A^ mice at the end of the disease but without extending survival [115]. Moreover, recent data report that P2X7 activation shows beneficial effects on microglia due to mitophagy and lysosomal biogenesis enhancement, but prolonged action leads to cell death [116]. Leaving aside this interesting target, all the information exposed in this review remarks that there are many issues in ALS waiting to be solved and mitophagy is clearly an exciting one of them.

## 6. Concluding Remarks

Current trends in life expectancy make ALS a public health challenge that needs to be faced. Mitochondrial homeostasis is clearly impaired in this disease, thus, autophagy and mitophagy dysregulation is one of the most studied issues on this pathology. On this basis, the use of autophagy and/or mitophagy enhancers may be appropriated in order to find an effective treatment for ALS. In the last years, the use of different autophagy modulators such as trehalose or rilmenidine has provided mixed results depending on the model, time administration, off-target effects, and other factors. Furthermore, rapamycin has reached clinical trials with no clear results. Far from being a failure, these results highlight the complex nature of these pathways and bring us closer to understanding ALS and the potential use of personalized medicine, mainly for the *SOD1*-ALS patients. Mitophagy and autophagy are clearly revealed to be fundamental in order to maintain NMJ in the short term, while in the long term, it likely must be combined with mitochondriogenesis and lysosomal function enhancers. On another front, autophagy and mitophagy appear to be critical pieces in glial cells towards reducing neuroinflammation and a promising strategy in the modulation of the disease progression.

Considering all this evidence, the discovery of small molecules able to modulate mitophagy and/or mitochondriogenesis gains the stage to provide critical future research tools. These molecules will play a crucial role in deciphering the significance of the relevant processes in health and disease. Moreover, mitophagy modulators emerge as new drug candidates to be incorporated in the race to find a cure for ALS and other rare diseases where mitochondrial dysfunction plays a relevant role.

## Abbreviations

ALS	Amyotrophic lateral sclerosis
AMBRA1	Autophagy and beclin 1 regulator 1
AMPK	AMP-activated protein kinase
ATP	Adenosine triphosphate
CCCP	Carbonyl cyanide m-chlorophenyl hydrazone
CNS	Central nervous system
CS	Citrate synthase
DAMPs	Damage-associated molecular patterns
fALS	Familiar ALS
FF motoneuron	Fast fatigable motoneuron
FR motoneuron	Fast resistant motoneuron
FUNDC1	FUN14 domain containing protein 1
*FUS*	Fused in sarcoma
GABARAPL1	Gamma-aminobutyric acid receptor-associated protein-like 1
IFN	Interferon type I
ISG	Interferon-stimulated genes
IRF3	Interferon regulatory factor 3
LAP	LC3-associated phagocytosis
LC3	Microtubule-associated proteins 1A/1B light chain 3B
LIR	LC3-interaction region
MARCH5	Membrane-associated ring finger 5 ubiquitin-ligase
MFN1	Mitofusin 1
MFN2	Mitofusin 2
MIRO1	Mitochondrial Rho GTPase 1
mTORC1	Mammalian target of rapamycin complex 1
MULAN	Mitochondrial ubiquitin-ligase activator of NF-kB
NBR1	Neighbor of BRCA1 gene 1
NDP52	Nuclear dot protein 52
NF-kB	Nuclear factor-kappa B
NMJ	Neuromuscular junction
*OPTN*	Optineurin
P2X7	P2X purinoceptor 7
PGC-1α	Peroxisome proliferator-activated receptor gamma coactivator 1-alpha
PINK1	PTEN-induced kinase 1
RLPK1	Receptor-like protein kinase 1
ROS	Reactive oxygen species
sALS	Sporadic ALS
SMCR8	Smith-Magenis syndrome chromosome region candidate 8
*SOD1*	Superoxide dismutase 1
TAX1BP1	TAX1 binding protein
*TBK1*	TANK-binding kinase 1
TDP-43	43 kDa transactive response DNA binding protein
TFAM	Mitochondrial transcription factor A
TIM	Translocase of the inner membrane
TLR3	Toll-like receptor 3
TOM	Translocase of the outer membrane
ULK1	Unc-51 like autophagy activating kinase 1
UPS	Ubiquitin-proteasome system
VDAC1	Voltage-dependent anion channel 1
